# The need for speed? Exploring the risks and benefits of pharmacological treatment for adult ADHD in prisons

**DOI:** 10.1080/13218719.2023.2260861

**Published:** 2024-01-15

**Authors:** Alana Grimley, Lorana Bartels

**Affiliations:** a Centre for Social Research and Methods, Australian National University, Canberra, Australia; b Criminology, Centre for Social Research and Methods, Australian National University, Canberra, Australia; c School of Law, University of Tasmania, Tasmania, Australia; d Canberra Law School, University of Canberra, Canberra, Australia

**Keywords:** attention deficit/hyperactivity disorder (ADHD), corrections, criminal justice system, dexamfetamine, diversion, lisdexamfetamine, methylphenidate, prison, stimulant, treatment

## Abstract

Attention deficit/hyperactivity disorder (ADHD) is overrepresented in prison populations globally. Although pharmacological treatment has generally been demonstrated to be an effective tool for managing ADHD symptoms in the general population, it remains controversial in the prison context. This is primarily due to concerns about the diversion and abuse of medication. To evaluate the impact of pharmacological ADHD treatment for adults who are or have been in prison, a narrative literature review was completed and seven international studies were identified. Further research into the use of stimulant medication in prisons, particularly within the Australian context, is recommended.

## Introduction

Attention deficit/hyperactivity disorder (ADHD) arises through a variety of environmental and genetic factors, resulting in hyperactive, impulsive and/or inattentive behaviours which are inconsistent with developmental age and interfere with functioning (American Psychiatric Association (APA), 2022). Typically seen as a childhood disorder, the diagnostic features of adult ADHD have only recently been included in the *Diagnostic Statistical Manual of Mental Disorders* (*DSM*; APA, 2022; Epstein & Loren, 2013). Global estimates suggest that approximately 5.9% of children and 2.5% of adults have ADHD (Faraone et al., 2021), while the prevalences in Australia are approximately 4% and 3%, respectively (Deloitte Access Economics, 2019).

As discussed in more detail below, there is extensive research indicating that people with ADHD have an increased risk of being involved in the criminal justice system (CJS), with international data suggesting that up to one quarter of prisoners have ADHD. In this context, the present paper reviews the literature on the impact of stimulant treatment of ADHD for adults who are or have been in prison, although we recognise that this should be used in the context of other treatment modalities, including psychoeducation and psychological treatment (Australian ADHD Guideline Development Group [AAGDG], 2022). The international evidence suggests there is a need to improve ADHD diagnosis and treatment practices within Australian prisons. Ultimately, given the paucity and uncertainty of current research in this field, we advocate for further research into the use of pharmacological treatment for ADHD in prisons.

## Literature review

### The medicalisation of ADHD

Although debating the reality of ADHD is beyond the scope of this paper, we acknowledge the sociological discourse, which interprets ADHD as the medicalisation and pharmaceuticalisation of a range of socially undesirable behaviours. *Medicalisation*, as described by Conrad (1975), means the process by which behaviours are defined as health issues, warranting medical expertise and treatment. *Pharmaceuticalisation* refers to how behaviours are considered in need of intervention (Conrad & Bergey, 2014). Childhood hyperactivity was initially medicalised under the diagnostic label of hyperkinesis (Conrad, 1975; Lane & Chong, 2019; Lusardi, 2019). Subsequently, ADHD has expanded rapidly, both in definition and global prevalence (Conrad & Potter, 2000; Lusardi, 2019). This expansion has been attributed to globalised pharmaceutical markets, the availability of online screening tools, community advocacy, the inclusion of adult symptomatology in diagnostic tools and, crucially, widespread employment of the *DSM* (Conrad & Bergey, 2014; Conrad & Potter, 2000; Lusardi, 2019).

The main criticism of the medicalisation of ADHD is the subjective nature of its diagnosis (Prosser et al., 2015). As a result, measures of the disorder’s prevalence and severity differ, according to the diagnostic tool in use (S. Young & Cocallis, 2021). The *DSM* and the *International Classification of Diseases* (*ICD*) are the two major sources of diagnostic information about ADHD (APA, 2022; World Health Organization (WHO), 2019). The *DSM* was developed by the American Psychiatric Association (APA) and largely serves psychiatrists in the United States (US), whereas the *ICD* was developed by the World Health Organization (WHO), serving a broader audience across disciplines, cultures and languages (American Psychological Association, 2009).

Whereas the *DSM* has included three subtypes of ADHD since the revised version of its third edition (APA, 1987), the *ICD-10* included hyperkinetic disorder to represent the combined subtype of ADHD, requiring symptoms of both hyperactivity and inattention for diagnosis (Conrad & Bergey, 2014; National Collaborating Centre for Mental Health, 2009). These differences between the diagnostic criteria have complicated comparative studies. For example, the assertion that hyperkinetic conduct disorder is a contributor to the relationship between ADHD and offending (see Mordre et al., 2011) may represent an association between the combined subtype of ADHD and offending (Rösler et al., 2004). However, both diagnostic tools have converged over time (American Psychological Association, 2009). In particular, the *ICD-11*, which came into effect on 1 January 2022, permits diagnosis of ADHD through symptoms of hyperactivity/impulsivity and/or inattention (WHO, 2019).

Despite the convergence of these diagnostic tools, sociological research has demonstrated the problematic nature of subjective categorisations of behaviour. A study of diagnostic decisions revealed that psychotherapists do not always adhere to diagnostic criteria (Bruchmüller et al., 2012). In vignettes which lacked key diagnostic criteria for ADHD according to both the *DSM* and the *ICD*, 17% of responding therapists made a false-positive diagnosis. Furthermore, boys were more commonly diagnosed with ADHD in the non-ADHD vignettes than girls. Bruchmüller et al. (2012) therefore suggest that subjective perceptions of ADHD may result in overdiagnosis.

Subjective interpretations of ADHD-related behaviours have also been shown to impact criminalisation (S. Young & Cocallis, 2021). For example, sociological perspectives have contributed to research into how ADHD can affect police interviews (Cunial et al., 2019; S. Young et al., 2013) and court participation (Freckelton, 2019). Despite these issues of subjectivity in identifying and interpreting ADHD, the disorder is acknowledged in the medical literature as real and clinically significant (Barkley, 2002) and is accepted as such in this paper.

### Treatment for ADHD

ADHD treatment includes pharmacological intervention, in the form of stimulant or non-stimulant medication, and psychological treatment, to improve pro-social competence. Stimulant medications are controlled substances which produce greater focus and calmness among individuals with ADHD (see Asherson et al., 2016). The principal stimulant medication options in Australia are lisdexamfetamine (Vyvanse^®^), dexamfetamine, and methylphenidate (Ritalin^®^, available in both short-acting and long-acting forms, and Concerta^®^) (see AAGDG, 2022; New South Wales (NSW) Government Health, 2018). The only ADHD-specific non-stimulant available in Australia is atomoxetine (Straterra^®^), although blood pressure medications such as guanfacine (Intuniv^®^) and clonidine (Catapres^®^) may also be prescribed, often for children with an ADHD diagnosis. According to Advokat and Scheithauer, ‘there is perhaps no other behavioral disorder than ADHD, for which drugs have so consistently proven acutely effective. Stimulants […] improve ADHD symptoms in about 70% of adults and 70–80% of children’ (2013, p. 2).

Expert consensus and guidelines indicate that stimulant medications should act as first-line treatment for ADHD (AAGDG, 2022; Kooij et al., 2019; National Institute for Health and Care Excellence, 2018; S. Young, Adamou, et al., 2011; S. Young, Gudjonsson, et al., 2018). This is due to their greater effect size compared to non-stimulant medications (Faraone et al., 2006; S. Young, Gudjonsson, et al., 2018). The *Australian Evidence-Based Clinical Practice Guideline* *for Attention Deficit Hyperactivity Disorder* – released by the Australian ADHD Professionals Association (AADPA) in late 2022 and endorsed by the National Health and Medical Research Council – recommends that methylphenidate, dexamfetamine and lisdexamfetamine should be offered as first-line treatment for adults whose ‘ADHD symptoms are causing significant impairment’ (AAGDG, 2022, p. 25). However, a recent systematic review investigating the impact of pharmacological treatment of adult ADHD has cast doubt on this assertion, suggesting that the positive effects of stimulant medication are negligible and difficult to assess due to limited data and the heterogeneity of results (Boesen et al., 2022). Although the use of stimulant medication for adult ADHD is recommended in the AADPA’s guidelines, the authors nonetheless express low confidence that the size of its effect as reported in current research is accurate (AAGDG, 2022).

Like any medication, the use of stimulants of this nature brings some risk. Common adverse events include mucosal dryness, headaches, loss of appetite, irritability and insomnia (NSW Government Health, 2018). In addition, the use of stimulant medication is associated with an increased risk of cardiovascular side effects, such as increased heart rate, stroke, myocardial infarction or sudden death in extreme cases, although the risk of major cardiovascular complications is minimal (Kooij et al., 2019). Accordingly, non-stimulant medications are preferable in the event of an adverse reaction to stimulants (S. Young, Gudjonsson, et al., 2018). In addition, non-stimulant medications are recommended when stimulants are ineffective and when a sustained 24-hour effect is desirable. Even long-acting stimulant medications, such as lisdexamfetamine, wear off after approximately 8 to 12 hours (depending on individual metabolism), whereas guanfacine lasts for up to 24 hours.

The use of stimulant medications for managing ADHD within prison remains controversial, due to the risk of drug-seeking behaviours that can result in treatment diversion and abuse (Ginsberg et al., 2013; Mattes, 2016; S. Young, Adamou, et al., 2011; S. Young, Gudjonsson, et al., 2018). Regardless, failure to address ADHD symptoms is particularly problematic, given that adults with ADHD are overrepresented in prisons (S. Young, Moss, et al., 2015).

### ADHD and offending

ADHD is associated with increased offending. International studies reveal that incarcerated people with ADHD exhibit earlier contact with the CJS and more prior offences than those without ADHD (S. Young & Cocallis, 2021; see also Dagistan et al., 2022; Moore et al., 2016; Rösler et al., 2004, 2009; S. Young, Wells, & Gudjonsson, 2011). Mohr-Jensen and Steinhausen’s (2016) meta-analysis of longitudinal studies revealed that individuals with childhood ADHD were two-to-three times more likely than those without ADHD to experience arrest, conviction and incarceration as adolescents and adults.

These offending patterns are partially explained by comorbid disorders. Mordre et al. (2011) suggest that ADHD is associated with increased risk of offending only when comorbid with conduct disorders, which are a common comorbidity. Furthermore, conduct disorder has been said to mediate the relationships between ADHD and substance use disorder (SUD; see further discussion below, and see Biederman et al., 2008; Lynskey & Hall, 2001) and between ADHD and offending (Mohr-Jensen & Steinhausen, 2016; Mordre et al., 2011). Although these findings may suggest that ADHD itself is not a contributor to offending, the disorder rarely exists without comorbidity and must be understood as it coexists with other psychiatric disorders.

Pharmacological treatment for ADHD has been shown to reduce risk of offending in the community. A longitudinal study tracking patient registers in Sweden revealed that people with ADHD were more likely to be convicted of a crime than those without ADHD (Lichtenstein et al., 2012); however, the risk of offending was reduced during treatment periods by 32% among men and 41% among women.

### ADHD and prison

Although estimates vary by country, there is consensus that ADHD is overrepresented in prison compared to the general population. Meta-analysis of international studies suggests that 21% of the prison population meets the diagnostic criteria for ADHD (S. Young, Moss, et al., 2015). However, this estimate is likely to be conservative, with male prison populations exhibiting prevalence rates of up to 45% (Dagistan et al., 2022; Ginsberg et al., 2010; Rösler et al., 2004; S. Young, Moss, et al., 2015) and female prison populations exhibiting rates of up to 30% (Edvinsson et al., 2010; Konstenius et al., 2015; Rösler et al., 2009; S. Young, Moss, et al., 2015). According to S. Young, Moss, et al. (2015), however, there is no statistically significant difference between the prevalence of ADHD among men and women in prison.

There is limited information on the incidence of ADHD in Australian prisons. Notably, the national survey of prisoner health does not refer to ADHD (Australian Institute of Health and Welfare, 2019). A 2016 survey in the Australian Capital Territory’s adult prison, which houses all men and women, found that 19% of participants screened positively for ADHD (J. T. Young et al., 2017). In a study of two male and two female prisons in New South Wales (NSW), 17% of participants met the full diagnostic criteria for ADHD – a significantly greater prevalence than in the general population (Moore et al., 2016).

Despite the high prevalence of ADHD among prisoners, the disorder often goes undiagnosed. The aforementioned study from NSW revealed that, among individuals who screened positively for ADHD, only 50% had received an ADHD diagnosis prior to participating in Moore et al.’s (2016) study and only 26% had previously been prescribed medication. These rates were even lower in a Scottish prison, where only 19% of respondents with ADHD had previously been diagnosed and just 16% reported having received pharmacological treatment (S. Young, González, et al., 2018). In a Swiss prison, 13% of participants were considered to potentially have ADHD, according to self-reports, yet only 2% of participants had a clinical diagnosis reported in their medical records (Baggio et al., 2022). None of the participants were receiving treatment during their detention.

Furthermore, adults with ADHD in prison are often impacted by multiple comorbidities. Up to 96% of studied prisoners with ADHD have at least one psychiatric comorbidity (S. Young & Cocallis, 2021). Rösler et al. (2004) noted that 64% of the prison population studied had at least two diagnosed psychiatric disorders, in addition to ADHD. International studies of ADHD prevalence suggest that SUD is a comorbidity in up to 100% of studied prison populations (Ginsberg et al., 2010; Rösler et al., 2009; S. Young, Sedgwick, et al., 2015). Crucially, comparing psychiatric comorbidity between incarcerated youths and adults with ADHD revealed that adults presented with greater comorbidity over time (S. Young, Sedgwick, et al., 2015). Understanding ADHD treatment in prisons may therefore be more complex amongst affected adults.

As a result of the associations between ADHD and both offending and imprisonment, ADHD generates significant costs to the CJS. In Australia, it was estimated in 2019 that the costs of crime and to the CJS, due to ADHD, were $307 million (Deloitte Access Economics, 2019). Within prisons, ADHD attracts further costs due to health needs. Individuals with ADHD in a Scottish prison required more visits with general practitioners, and with mental and physical health nurses, than those without ADHD (S. Young, González, et al., 2018).

### Pharmacological treatment for ADHD in prison

Treating ADHD within prisons presents unique challenges. In particular, the use of stimulant medications in prisons is controversial due to their status as controlled substances, which may increase the potential for abuse and diversion (S. Young & Cocallis, 2021). This is especially of concern among populations which exhibit ADHD and SUD. There is a broad range of research attempting to determine how ADHD is related to substance use. Illicit psychostimulant use has been hypothesised as a form of self-medication for ADHD symptoms (Kaye et al., 2013), as illicit drug users with ADHD in prisons often demonstrate a preference for amphetamines (Ginsberg et al., 2010; Ginsberg & Lindefors, 2012; Konstenius et al., 2014; Rösler et al., 2009; S. Young, González, et al., 2020). However, although ADHD is prevalent among illicit psychostimulant users (Kaye et al., 2013), in the absence of conduct disorder there is some evidence to suggest that ADHD itself is not associated with increased risk of SUD (Lynskey & Hall, 2001). Furthermore, stimulant medications present minimal risk for developing SUD and may instead serve as a protective factor (Biederman et al., 2008; Hammerness et al., 2017).

Non-stimulant medications, while not as effective as stimulant medications, have also been considered for use within the prison environment due to their potential to reduce anxiety and exert a sedating effect (Mattes, 2016). A retrospective study exploring non-stimulant treatment in a correctional community centre suggested that individuals with suspected ADHD and comorbid SUD exhibited statistically significant global improvement (Bastiaens et al., 2019).

Given the listed concerns regarding stimulant medication distribution in prisons (Ginsberg et al., 2013; Scott et al., 2016; S. Young, Adamou, et al., 2011; S. Young, Gudjonsson, et al., 2018), a stringent treatment protocol was developed by the University of Massachusetts Correctional Health (Appelbaum, 2009, 2011; Scott et al., 2016). The protocol prioritised non-stimulant treatment. To receive stimulant medications, individuals were required to provide evidence of prior ADHD diagnosis, severe functional impairment in prison life, adverse or limited response to non-stimulants and proof of attendance at recommended psychological interventions (Appelbaum, 2009, 2011). As a result, less than 1% of male prisoners in Massachusetts were eligible to receive stimulant medications (Appelbaum, 2011). Although beneficial in ensuring that treatment abuse was limited, this approach also impacted treatment efficacy.

Consensus documents developed by the United Kingdom (UK) ADHD Partnership emphasise that such harsh approaches to preventing abuse are unnecessary, as once-daily, long-acting stimulant medications significantly reduce abuse risk (National Institute for Health and Care Excellence, 2018; S. Young, Gudjonsson, et al., 2018). In Australia, these include: Osmotic-Release Oral System Methylphenidate (OROS-MPH); long-acting methylphenidate; and controlled-release lisdexamfetamine (NSW Government Health, 2018). Furthermore, prison dispensaries are already equipped to safely distribute controlled substances (S. Young, Adamou, et al., 2011) and expert opinion suggests that diversion risk is greater for anti-depressant and anti-psychotic medications (S. Young, Gudjonsson, et al., 2018). Consensus among advocates and experts from Europe therefore maintains that stimulant medications are the best first-line treatment for ADHD (Kooij et al., 2019; S. Young, Adamou, et al., 2011; S. Young, Gudjonsson, et al., 2018), in contrast with the approach proposed by the Massachusetts protocol (Appelbaum, 2009, 2011). The Australian guideline recommends that stimulant distribution frameworks align with those utilised for the distribution of other controlled substances within prisons (AAGDG, 2022).

In Australia, long-acting stimulant medications for ADHD are subsidised on the Pharmaceutical Benefits Scheme (PBS) and are thus generally accessible and affordable, although more so for those diagnosed before the age of 18. The *Guiding Principles for Corrections in Australia*, which all states and territories are expected to utilise when developing corrections policies, advocate that ‘[p]risoners are provided a standard of health care equal to services available in the community that meet their individual physical health, mental health and social care needs fostering continuity of care between custody and the community’ (Corrective Services Administrators’ Council, 2018, p. 20). As stimulant medications are readily available in Australia, and as their use has increased in the general population over time (Prosser et al., 2015), the same standard of care should be expected within prison, enabling the prescription of stimulant medications for ADHD (as well as the provision of other treatments, such as educational and psychological treatment responses). However, evaluation of treatment efficacy relies upon evidence that improved outcomes compare favourably against the risk of diversion, abuse and possible adverse side effects. Therefore, the following narrative review examines the evidence on adult ADHD treatment in prison, in order to explore the outcomes of pharmacological interventions to date.

## Methodology

A literature search was conducted by utilising keywords in a series of databases, including Google Scholar, Ovid, PubMed and ProQuest, to identify literature in the cross-section of criminology, psychology and medicine. Key search terms included: ‘attention deficit hyperactivity disorder’, ‘ADHD’, ‘adult’, ‘prison’, ‘treat*’, ‘medicat*’, ‘stimulant’ and ‘non-stimulant’. Literature was excluded if participants were under the age of 18, psychological treatment was the primary focus of the study or the prison context was not discussed. No date range was imposed for the initial literature search; however, investigation revealed that the first clinical trial on this topic occurred in 2012. Seven papers are discussed in this review, spanning 2012 to 2023, including pilot studies, clinical trials and a cohort study.

## Findings: what is the impact of ADHD treatment for adults who are or have been in prison?

Seven studies were identified which investigated the impact of pharmacological treatment for adults with ADHD who were or had been in prison (see Table 1). These trials largely investigated stimulant medications, measuring change in outcomes such as ADHD symptoms, functional impairment, recidivism and critical incidents in prison. Five of these studies took place in Sweden and the remaining two were undertaken in the UK.

**Table 1.** Identified studies investigating pharmacological ADHD treatment for adults during or after imprisonment.

Study	Sample characteristics	Diagnostic tools	Treatment	Measures
Ginsberg and Lindefors (2012) Sweden	Adult males, 21–61 years old, incarcerated in Norrtälje Prison *n =* 30	Hare Psychopathy Checklist-Revised Structured Interview for: *DSM-IV* consistent ADHD *DSM-IV* Axis-I Disorders *DSM-IV* Axis-II Disorders Patient Questionnaire	OROS-MPH *n =* 15 Placebo *n =* 15 OROS-MPH open-label extension *n =* 30	The experimental group achieved a clinically significant reduction in ADHD symptoms. ADHD symptoms and global functioning continued to improve during the open-label extension. Adult ADHD Self-Report Scale (ASRS) Conner’s ADHD Rating Scale: Observer Screening Version (CAARS-O:SV) Clinical Global Impressions Scale (CGI-S) Global Assessment of Functioning Scale (GAF) Reported adverse events
Ginsberg et al. (2012) Sweden	As above	As above	As above	In addition to the above results, working memory, cognition and motor activity measures improved within the initial trial and continued to improve or remained stable during the open-label extension. As above and: Conner’s Continuous Performance Test II Institutional Behaviour QbTest Quality of Life Inventory (QOLI) Weschler Adult Intelligence Scale (WAIS)-III ○ Similarities WAIS ○ Digit Span ○ Span Board
Ginsberg et al. (2015) Sweden	Participants who completed the final 52-week assessment in Ginsberg and Lindefors (2012) • One year post-trial, *n =* 24 • Three years post-trial, *n =* 20	As above	Naturalistic follow-up; continuation of treatment was contingent on a number of factors OROS-MPH • One year post-trial, *n =* 20 • Three years post-trial, *n =* 15 • No ADHD medication One year post-trial, *n =* 4 • Three years post-trial, *n =* 5	Observer-rated and self-reported reductions in ADHD symptoms were maintained in all completing participants across the one and three-year follow-up assessments, with a large effect size. Improvements in all other measures of psychosocial functioning at the conclusion of the 52-week trial were maintained at both follow-ups. Alcohol and drug use remained below the threshold of substance-related problems according to their respective scales among all completers at the one- and three-year follow-ups. Alcohol Use Disorders Identification Test ASRS CAARS-O:SV CGI-S Drug Use Disorders Identification Test GAF QOLI
Konstenius et al. (2014) Sweden	Incarcerated males, 18 to 65 years old, recruited from three medium security Swedish prisons • *n =* 54	Addiction Severity Index • Adult ADHD Self-Report Scale • Conner’s Continuous • Performance Test • Structured Clinical • Interview for *DSM-IV* • Axis I and Axis-II • Disorders • WAIS-II: Short Form • Wender-Utah Rating • Scale	OROS-MPH • *n =* 27 • Placebo • *n =* 27	Results demonstrated a clinically significant reduction in ADHD symptoms among the experimental group, greater retention to treatment and fewer drug-positive urine tests. CAARS-O:SV CGI-S Craving for Amphetamine Scale Outcome Questionnaire Reported adverse events Time to Relapse Urinalysis
Chang et al. (2016) Sweden	Prisoners released from Swedish Prison and Probation Services between 2005 and 2010 • *n =* 22, 275	*ICD9/10* criteria for ADHD, recorded on the National Patient Register	Anti-psychotics, psychostimulants and medication for addictive disorders	Within-individual analysis revealed that stimulant users committed 42.8 fewer violent reoffences per 1000 person years during treatment. National Crime Register: post-release conviction for violent crime
Asherson et al. (2016) UK	Incarcerated males, 18 to 30 years old, from an English prison • Baseline: • *n =* 121 • 12-week assessment: • *n =* 72	Barkley Adult Current ADHD Questionnaire • Clinical Interview for *DSM-IV*	OROS-MPH	Results demonstrated a statistically significant difference in clinical ADHD symptoms, with a large effect size. Adverse Events Scale (AES) CAARS-O:SV Engagement with education Modified Overt Aggression Scale (MOAS) Recorded Critical Incidents Wender–Reimherr Adult Attention Deficit Disorder Scale (WRAADS)
Asherson et al. (2023) UK	Incarcerated males, 16 to 25 years old, from an English and Scottish prison • *n =* 200	Barkley Adult ADHD • Rating Scale Diagnostic Interview for ADHD in Adults, *DSM-IV* (DIVA V2.0)	OROS-MPH • Placebo	Both trial arms experienced a reduction in ADHD symptoms. The experimental group evidenced a slightly greater reduction in symptoms from baseline compared to the placebo group at a small, non-significant level. The same pattern of small, non-significant improvements in the experimental group were observed across secondary measures. AES Affective Reactivity Index – Self-Report Behaviour Report Cards – Prison Officers & Education Staff Brief Symptom Inventory CAARS-O:SV Clinical Global Impression – Therapeutic Response and Severity Scores Clinical Outcomes in Routine Evaluation – Self-Report CORE Outcome Measures Engagement with education Maudsley Violence Questionnaire Mind Excessively Wandering Scale Mini-International Neuropsychiatric Interview 7.0.1 MOAS – Prison Staff & Education Staff Recorded Critical Incidents WRAADS

Ginsberg and Lindefors (2012) completed a double-blind, randomised controlled trial (RCT) to test the efficacy and safety of OROS-MPH among incarcerated men with ADHD – the first study of its kind. The sample comprised 30 men in a high-security prison in Sweden. This trial offered a holistic treatment approach, as participants were also offered educational, vocational and treatment programmes based on assessment of their individual needs. Within the initial five-week trial, 87% of participants in the experimental condition achieved a clinically significant reduction in ADHD symptoms, defined as a 30% reduction in symptoms. Despite the small sample size, this reduction produced a large effect size, with a Cohen’s *d* score of 2.17. The experimental group also significantly outperformed the placebo group on self-reported and assessor-rated measures of psychosocial functioning. It is noteworthy that the placebo group, who were offered the treatment drug during a 47-week open-label extension after the completion of the RCT, demonstrated significant improvement on all measures, although to a lesser extent.

Long-term outcomes from Ginsberg and Lindefors (2012) were later reported in Ginsberg et al. (2012) using neuropsychological tests, self-reported quality of life and reports of institutional behaviour. The study measured unique functional outcomes rarely explored in other trials, demonstrating that individuals taking OROS-MPH experienced a significant improvement in working memory, cognition and motor skills from baseline, with peak effect achieved at 16 weeks and results maintained to the completion of the trial. Furthermore, 80% of the participants engaged in at least one programme during the trial – a favourable outcome that the authors note may not have been possible without the steadying effects of ADHD medication (Ginsberg et al., 2012).

Participants who provided data at the completion of the 52-week trial were invited to participate in clinical follow-ups at one and three years after the trial concluded (Ginsberg et al., 2015). This naturalistic study demonstrated that – among all completers, whether they continued taking medication or not – observer-rated and self-reported ADHD symptoms remained significantly lower than baseline results at both follow-ups. However, participants who continued to take medication had significantly fewer observer-rated and self-reported ADHD symptoms and less alcohol and drug use compared to those no longer taking medication. This study also reported on outcomes for participants released from prison in the follow-up period, noting a favourable employment rate. In addition, reoffending rates within three years of release were lower for participants than other men released from prison in Sweden with the same principal offence during the study period. However, the authors acknowledged that the small study population in the naturalistic follow-up limits interpretation of these outcomes. Furthermore, while this trial demonstrated significant short-term efficacy and improved long-term functional outcomes, the control group was not retained through the open-label extension or naturalistic follow-up, limiting comparison of outcomes between subjects beyond the initial five-week trial.

Although efficacy is quantitively evident in the above studies, evaluating the safety of pharmacological treatment requires investigation of the side effects as well as diversion and abuse risk. The most commonly reported adverse events during this trial included ‘abdominal discomfort, headache, mucosal dryness, depressed mood, loss of appetite, anxiety, diarrhoea, sweating, interrupted sleep and fatigue’ (Ginsberg & Lindefors, 2012, p. 71). Although the participants rated these events as mild-to-moderate and none withdrew from the study as a result, quantitative analysis cannot reveal whether or not the relative pain of these symptoms was amplified within a prison environment. Like many medications, however, these side effects may also decrease over time.

With regard to treatment diversion and abuse, Ginsberg and Lindefors (2012) attempted to maintain external validity by retaining a population which evidenced psychiatric comorbidity, including antisocial personality, autism spectrum and anxiety disorders. Notably, all participants met the diagnostic criteria for conduct disorder and SUD, presenting a risky treatment population with regard to potential substance abuse. Participants were therefore excluded if they had abused substances in the three months before the trial commenced. To further limit diversion and abuse risk, most of the participants were moved to the ADHD wing of the prison throughout the trial. The ADHD wing provided a highly structured and supportive environment in which study participants and prison staff were educated about ADHD by the researchers. Although the desired effect of this environment was achieved given that no substance misuse was reported during the trial, one participant revoked consent due to dissatisfaction with the ADHD wing (Ginsberg & Lindefors, 2012). This brings into question whether dispensary logistics alone can prevent diversion and abuse or whether more extreme measures, including segregating stimulant medication recipients, are necessary.

A more recent randomised placebo-controlled trial by Konstenius et al. (2014) utilised a larger sample, recruiting 54 men from 3 medium-security prisons in Sweden. Also examining the effects of OROS-MPH, the trial began 2 weeks before the participants’ release from prison. This study was designed to target participants with comorbid ADHD and amphetamine dependence in order to examine the safety and efficacy of OROS-MPH to treat ADHD symptoms and prevent drug relapse. The trial ran for 24 weeks and required participants to abstain from using any illicit substances for 2 weeks prior to its commencement. Compared to earlier Swedish trials, which titrated the medication dose from 18 mg to 72 mg (Ginsberg et al., 2012; Ginsberg & Lindefors, 2012), this trial used a significantly higher dose of 180 mg.^1^ However, this dose is unsurprising given that participants in the first Swedish trial reached an average daily dose of 108 mg during the open-label extension, and trial completers who continued using medication reached an average daily dose of 144 mg (Ginsberg et al., 2015; Ginsberg & Lindefors, 2012). The high dose in this population could be attributable to an increased tolerance to stimulants due to prior substance use, or increased levels of executive function required upon release from a structured environment like prison (Ginsberg et al., 2015). Within the experimental group, 65% of participants experienced a clinically significant reduction in self-reported ADHD symptoms; however, 27% of participants in the placebo group also experienced a clinically significant reduction in symptoms (Konstenius et al., 2014).

As Konstenius et al.’s (2014) study occurred outside of a prison environment, the risk of drug diversion and abuse was not such a concern. However, the amphetamine dependence-related outcomes indicate how OROS-MPH can serve as a protective factor against substance abuse. Participants in the experimental condition demonstrated significantly greater treatment retention, fewer positive urine tests, a longer time until a first positive urine test and less reported craving for amphetamines. Nevertheless, the participants experienced a similar suite of adverse events as those reported in prison. These were considered only mild-to-moderate and included headaches, abdominal discomfort, sleep problems and appetite loss. More serious but less commonly reported symptoms included high blood pressure and heart palpitations, leading to a reduction in treatment dose for two participants. After the trial concluded, 52% of the participants from the experimental group and 33% from the control group respectively continued or initiated treatment. Although no qualitative measures of programme satisfaction were taken, this suggests that – among individuals with ADHD and comorbid amphetamine dependence re-entering society – stimulant treatment is sufficiently beneficial to seek or adhere to treatment. Furthermore, Konstenius et al.’s (2014) study provides a causal link between medical stimulant treatment and reduced desire for amphetamines, further suggesting that stimulant medication may serve as a protective factor against substance abuse for individuals with ADHD.

In order to observe the effect of pharmacological treatment on recidivism, Chang et al. (2016) investigated patterns of violent reoffending among individuals released from prison in Sweden during treatment and non-treatment periods. They examined the effect of a variety of psychotropic medications – described as medications used to treat mental disorders – by linking data from the Swedish Prison and Probation Service, the Prescribed Drug Register and the National Patient Register. Treatment was classified as a time-varying exposure, comparing treatment and non-treatment periods. A treatment period was considered the time between two prescriptions, unless they were more than three months apart, as psychotropic medications are dispensed for up to three months at a time in Sweden. This provided a way to interpret adherence but could not control for it completely. Regarding the effect of stimulant medications, within-subjects analysis found that, during treatment periods, individuals committed 42.8 fewer offences per 1000 person years, and between-subjects analysis revealed that treated individuals committed 33.9 fewer offences. Although not all individuals who were dispensed stimulant medications had an ADHD diagnosis, the statistical significance of these results remained when examining only diagnosed individuals. Given that imprisonment is commonly associated with subsequent recidivism, this study would have benefited from a control population who did not use any medication in order to determine differences in reoffending patterns.

Although the above studies from Sweden provide valuable insight into the efficacy of psychostimulant treatment for ADHD, the context of the Swedish justice system is not globally generalisable (Lichtenstein et al., 2012). However, similar prison trials have also been conducted in the UK. Asherson et al. (2016) completed an open-label pilot study, titled CIAO-I, investigating the impact of OROS-MPH (Concerta XL^®^) on aggression in prison, as measured by adjudications (i.e. reports made by prison staff about undesirable prisoner behaviours). This study was the first of its kind in the UK, aiming to estimate a treatment effect size, in order to inform a future clinical trial. Despite observing reductions in the number of adjudications, Asherson et al. (2016) could not draw meaningful conclusions about the contribution of OROS-MPH, due to the low number of adjudications delivered. However, they observed a statistically significant difference in ADHD symptoms, with a large effect size. As this trial was not placebo-controlled, they suggest that the within-subjects analysis overestimated the effect size, as placebo subjects have also experienced a reduction in symptoms in previous trials. Therefore, based on results observed in previous studies, the authors attributed 20% of the observed effect on ADHD symptoms to the treatment medication. They also found that the participants demonstrated a preference for lower doses, with 34% titrating to a stable dose of 36 mg per day. These findings anecdotally suggest that the participants did *not* exhibit drug-seeking behaviours, but rather titrated to a dose which maximised treatment response and minimised side effects (Asherson et al., 2016).

Asherson et al.’s (2016) trial was the first of the examined studies to report qualitative outcomes. At baseline, many participants reported emotional instability and had several reports of disruptive behaviour, resulting in adjudications. After treatment, participants not only reported reduced ADHD symptoms, more emotional stability and reduced adjudications but were able to hold jobs within prison and plan for release. The participants also valued the weekly follow-up sessions to discuss symptoms and treatment responses, demonstrating the benefits of gathering qualitative data.

The positive but inconclusive outcomes of Asherson et al.’s (2016) trial informed the approach taken in a subsequent clinical trial, CIAO-II (Asherson et al., 2019). This RCT similarly explored the impacts of OROS-MPH, focusing on ADHD symptoms as the primary outcome measure. The research design for CIAO-II aimed to address the concerns which emerged during CIAO-I, primarily those relating to treatment adherence and the substantial rate of attrition. The most notable changes made in response to the results of CIAO-I included a reduction in titrated dose, from 90 mg to 72 mg, closer monitoring of adverse effects and the inclusion of a broader range of investigator-rated, self-rated and prison-staff-rated outcome measures, in addition to prison records (Asherson et al., 2019, 2023). This study is therefore well positioned to provide more conclusive evidence of the impact of OROS-MPH on adults with ADHD in prisons.

Unlike the majority of the studies cited above, CIAO-II produced ‘robustly neutral’ results (Asherson et al., 2023, p. 16); ADHD symptom severity declined in both treatment arms, but while the experimental group exhibited a greater reduction in symptoms, the effect was small and non-significant. The experimental and placebo arms demonstrated poor adherence, but post hoc analysis demonstrated that similarly non-significant results were observed among adherent participants. Post hoc analysis also considered final titrated dose, self-reported drug use, comorbid disorders, diagnostic certainty and childhood trauma, none of which could suggest an explanation for the medication’s lack of effect. The authors emphasise that maximising external validity, by including a population with a history of psychiatric comorbidity and drug use, may have limited interpretation of the effect of OROS-MPH. Future research might consider investigating stimulant-naïve participants. The authors also note that placebo effects may impact results more uniquely in prison environments by introducing meaningful caregiving interactions in a setting where they are typically lacking. Individuals may also be more attuned to observation (the so-called Hawthorne effect) within a prison environment, impacting their behaviour. This study also had a number of limitations, including the lack of supervised drug screening (only self-report measures were used), high attrition rates and the low response rates in relation to the optimal treatment dose.

## Conclusion

The studies examined in this paper suggest that pharmacological treatment for ADHD is associated with numerous beneficial outcomes, both within prisons and after release. These outcomes include reduced ADHD symptoms, improved cognitive functioning and working memory, lower recidivism rates and reduced craving for amphetamines (Asherson et al., 2016; Chang et al., 2016; Ginsberg et al., 2012, 2015; Ginsberg & Lindefors, 2012; Konstenius et al., 2014; Lichtenstein et al., 2012). By contrast, the most recent study produced negligible impacts on ADHD symptoms and secondary outcome measures (Asherson et al. 2023). In addition, all seven identified trials indicated that treatment diversion and abuse is either insubstantial or preventable. This suggests that further trials can and should be safely undertaken in prison settings, in order to explore potential confounders and produce greater confidence in the results.

Nonetheless, the risks must be acknowledged. Although the studies examined in this paper recorded adverse events as mild-to-moderate, Asherson et al. (2016) suggested that side effects may be amplified within prison. There is a dearth of qualitative research in the field (cf. Asherson et al., 2016), with few trials including evaluations of adverse events and their relative pains, in contrast with the benefits of treatment – although some inferences can be drawn from attrition and/or retention rates (Asherson et al., 2016; Konstenius et al., 2014).

Another distinct gap in the research is the failure to investigate how women in prison respond to pharmacological ADHD treatment. Expert consensus suggests that stimulant medications are not suitable for women who are pregnant or breastfeeding, and appetite loss can exacerbate eating disorders, which are already prevalent within the female prison population (S. Young, Adamo, et al., 2020). Given that ADHD is similarly prevalent among men and women in prison (S. Young, Moss, et al., 2015), there is a clear need for further research on the effects of treatment within female prison populations, as different patterns of comorbidity may mediate the effects of treatment discussed here.

As these studies have only explored stimulant treatment in prisons in Sweden and the UK, further exploration is clearly needed to determine their application in the Australian context. The Australian clinical guideline acknowledges that further research is needed into models of care for ADHD (AAGDG, 2022). It is worth noting that the Australian National Disability Insurance Scheme does not provide support for individuals with ADHD in the general population, unless there is a relevant comorbidity. In addition, people in prison are not eligible for support under the *National Disability Insurance Scheme Act 2013* (Cth), s. 7.25. Furthermore, Vyvanse^®^ is currently the only long-acting stimulant medication subsidised through the PBS for those diagnosed with ADHD as adults (Hunt, 2021). All of these issues limit the ability of people affected by ADHD and its comorbidities in Australia, especially those in prison, to access the necessary supports and appropriate medication.

There is significant scope for the correctional environment to play a key role in identifying and treating underlying ADHD and better recognising its role in the substance use of incarcerated people. The new Australian ADHD guideline makes a number of recommendations relevant to the Australian justice system, including the following:

- organisations that provide services to people from high-risk groups could consider systematic screening for ADHD (Rec 1.2.2);
- screening and assessment processes should be established to identify the presence of ADHD and co-occurring conditions among people entering the CJS (Rec 6.1.1);
- custodial staff and others in the CJS should receive ADHD awareness training (Rec 6.1.2);
- prisoners with ADHD should have a comprehensive multi-agency integrated and coordinated care plan, with close coordination between the criminal justice, mental health and disability organisations, and at all transition points (Rec 6.1.6); and
- prisons should be resourced to enable the identification and treatment of people with ADHD (Rec 6.1.7). (AAGDG, 2022)

Introducing diagnosis and treatment practices for ADHD in Australian prisons would also require further insight into the needs of Aboriginal and Torres Strait Islander people with ADHD. In one of the few Australian studies regarding ADHD prevalence in prison, those who screened positively for ADHD were more likely to have a younger age at first contact with the CJS, report parental incarceration and identify as Indigenous (Moore et al., 2016). As Aboriginal and Torres Strait Islander people are more likely to experience earlier CJS contact and parental incarceration than non-Indigenous people, determining how much of this pattern is due to intergenerational trauma, systemic racism and/or the ongoing hyperincarceration of Indigenous people, as opposed to ADHD, is difficult and remains a gap in the literature. Furthermore, research suggests that attitudes to ADHD treatment among Aboriginal and Torres Strait Islander people tend to prioritise respect for the community and the rights of the child (Loh et al., 2016, 2017). This can translate to attitudes which may only support medication if used in conjunction with culturally appropriate social treatment programmes (Ghosh et al., 2015; Loh et al., 2016, 2017). Further investigation into the prevalence and treatment of ADHD among Aboriginal and Torres Strait Islander detainees would best be guided by the principles outlined in the report *Working Together: Aboriginal and Torres Strait Islander Mental Health and Wellbeing Principles and Practice* (Dudgeon et al., 2014) and in the ADHD guideline in the AAGDG (2022, [6.2]) which focuses on Aboriginal and Torres Strait Islander peoples.

The *Standard Minimum Rules for the Treatment of Prisoners (the Nelson Mandela Rules)* (United Nations, 2015) assert that imprisonment is punishment in and of itself and that prisons should not aggravate suffering but aim towards rehabilitative goals. Neither the risk of drug diversion and abuse nor the adverse side effects associated with stimulant medications appear sufficient to discount the potential benefits for adults with ADHD in prison; this will ensure that Australian correctional agencies are able to comply with their obligations under the *Guiding Principles for Corrections in Australia* (Corrective Services Administrators’ Council, 2018) when dispensing ADHD medication.

More research is required in the Australian context – especially with Aboriginal and Torres Strait Islander peoples and women. It is recognised that the prison setting is a complex environment for undertaking research and that incarcerated people with ADHD may be particularly challenging to engage in research. However, it can defensibly be inferred that these individuals have experienced severe consequences from having ADHD, which has commonly gone unidentified and untreated. Arguably, they should therefore be prioritised for intervention. The studies examined in this paper suggest that pharmacological treatment for ADHD both during and after imprisonment may benefit diagnosed individuals, as well as the justice system and communities more generally.

## Footnotes

1 We note that, for ADHD, 72 mg is the maximum dose generally given, but participants with SUD are generally able to tolerate and may even need higher doses in order to obtain a satisfactory reduction in their ADHD symptoms (AAGDG, 2022).
